# Systematic Review of Risk factors and Incidence of Acute Kidney Injury Among Patients Treated with CAR-T Cell Therapies

**DOI:** 10.1016/j.ekir.2021.02.013

**Published:** 2021-02-17

**Authors:** Swetha R. Kanduri, Wisit Cheungpasitporn, Charat Thongprayoon, Tananchai Petnak, Yi Lin, Karthik Kovvuru, Sandhya Manohar, Kianoush Kashani, Sandra M. Herrmann

**Affiliations:** 1 Department of Medicine, Ochsner Medical Center, New Orleans, Louisiana, USA; 2 Division of Nephrology and Hypertension, Department of Medicine, Mayo Clinic, Rochester, Minnesota, USA; 3 Division of Pulmonary and Pulmonary Critical Care Medicine, Faculty of Medicine, Ramathibodi Hospital, Mahidol University, Bangkok, Thailand; 4 Division of Pulmonary and Critical Care Medicine, Department of Medicine, Mayo Clinic, Rochester, Minnesota, USA; 5 Division of Hematology, Department of Medicine, Mayo Clinic, Rochester, Minnesota, USA

Immunotherapy has grown significantly in the management of hematological and solid organ tumors. In-depth knowledge of T-cell pathways has led to the development of adoptive cell transfer techniques and subsequent evolution of chimeric antigen receptor T-cell (CAR-T) therapies.^1^ CAR-T cells are biologically engineered cells with a CAR receptor that recognizes a tumor antigen. T-cell receptors are equipped with extracellular domain and variable costimulatory domains yielding divergent T-cell response.^2^ Along with their utility among refractory B cell lymphomas, adult diffuse large B-cell lymphoma (DLBCL), and pediatric acute lymphoblastic leukemia (ALL), CAR-T therapies are now being explored in the management of solid organ tumors and multiple myeloma.^3^

Cytokine release syndrome (CRS), macrophage activation syndrome (MAS)/ hemophagocytic lymphohistiocytosis (HLH), and immune effector cell−associated neurotoxicity syndrome (ICANS) are among the most alarming complications of CAR-T cells.S1 The incidence of acute kidney injury (AKI) reported in the literature among patients with CAR-T therapies varies from 5% to 33%.S1−S22 The mechanism of AKI in patients with CAR-T cell therapy is not completely understood. However, it is proposed that AKI could be secondary to inflammation associated with cytokine release, potentially leading to acute tubular injury.S19 Even though high-grade CRS is associated with AKI,S18 the correlation between severe AKI requiring renal replacement therapies (AKI-RRT) and high grades of CRS is not widely known. We conducted a current systematic review of literature and meta-analysis of articles reporting the incidence of AKI among patients receiving CAR-T therapies and the correlation between severity of CRS and AKI-RRT incidence.

## Materials and Methods

We followed the Preferred Reporting Items for Systematic Reviews and Meta-Analyses (PRISMA)S23 statement in conducting systematic review. EMBASE, Cochrane, and Ovid MEDLINE databases were systematically searched from database inception through May 2020. Full details are provided in the Supplementary Material.

## Results

**Figure 1:**
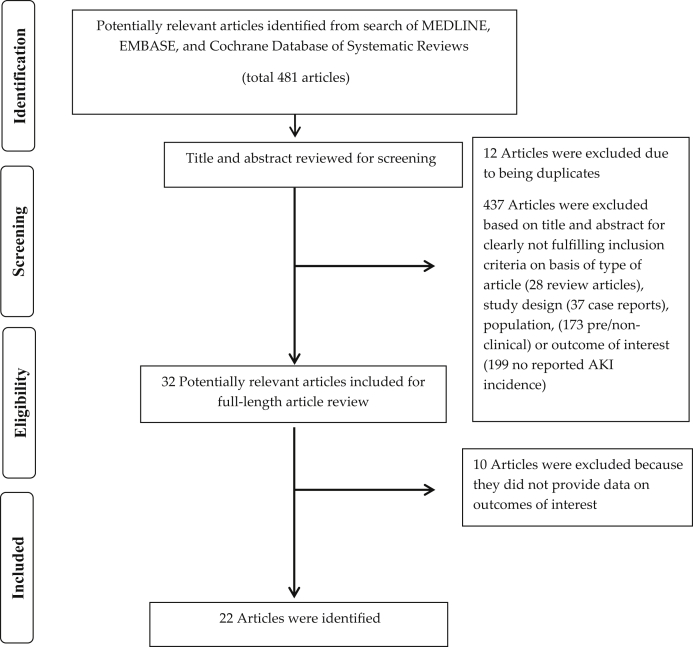
Preferred Reporting Items for Systematic Reviews and Meta-Analyses (PRISMA) flow diagram for study selection.

A total of 481 potentially relevant articles were identified and screened. In all, 32 articles were assessed in detail, of which 22 cohort studies S1−S22 with 3376 patients (Table 1) were included in our systematic review (Figure 1).

**Table 1:** Main characteristics of studies included in this meta-analysis of AKI incidence and mortality among patients treated with CAR-T cell therapy

Study	Year	Age	Indications for CAR-T therapy	Total number of patients (n)	AKI definition	AKI incidence/ severe AKI	Cause of AKI/biopsy	CRS grading	Baseline GFR	CRS with AKI vs. CRS without AKI	Mortality
Sauter *et al.*S2	2014	61 yr (34−68 yr)	DLBCL B-NHL	6	Not defined	Overall AKI, 1/6 = 16.6%	NA	Non-severe CRS, 1/2 = 50% Severe (grade 4 CRS), 1/2 = 50%	NA	Overall CRS, 2/6 = 33.3% No AKI mortality defined	NA No AKI mortality defined
Lee *et al.*S3	2014	5−27 yr (range)	Refectory B-ALL, NHL	21	Not defined	Overall AKI, 1/21 = 5%	NA	CRS grade 1, 8/16 = 50% CRS grade 2, 2/16 = 12.5% CRS grade 3, 3/16 = 18.75% CRS grade 4, 3/16 = 18.75%	NA	Overall CRS, 16/21 = 76%, No AKI CRS defined	NA
Kochenderfer *et al.*S4	2015	49 yr (range 30−68 yr)	DLBCL, CLL, B CELL lymphoma	15	Not defined	Overall AKI, 1/15 = 7%	NA	NA	NA	NA	Overall, 30- day mortality, 1/15 = 7% No AKI mortality defined
Ali *et al.*S5	2016	50 yr (range not reported)	MM	12	Not defined	Overall AKI, 2/12 = 16.6%	NA	CRS grade 3 and 4, 2/4 = 50%	NA	Overall CRS, 4/12 = 33.3%	NA
Fitzgerald *et al.*S1	2017	Median 11 yr (range 5−22 yr)	B-ALL	39	KDIGO	Overall AKI, 18/39 = 46% Stage 1, 9/39 = 23% Stage 2, 6/39 = 15% Stage 3, 3/39 = 8% None required RRT	NA	CRS grade 1, 2/36 = 6% Grade 2, 16/36 = 44.4% Grade 3, 7/36 = 19.4% Grade 4, 11/36 = 30.5%	NA	Overall CRS, 36/39 = 92%	NA
Locke *et al.*S6	2017	49 yr (range 29−68 yr)	DLBCL	7	Needing RRT	AKI needing RRT, 1/7 = 14%	NA	CRS grade 1 and 2, 5/6 = 83% CRS grade 3, 0 CRS grade 4, 1/6 = 17%	NA	Overall CRS, 6/7 = 86% AKI with CRS, 1/6 = 16%	9-mo Overall mortality, 4/7=57% No AKI mortality defined
Hay *et al.*S7	2017	54 y (median 20−73 yr)	ALL CLL NHL	133	Not defined	Overall AKI, 3/133 = 2.25% (AKI reported in only grade 4 CRS patients) AKI needing RRT, 1/133 = 0.75%	NA	CRS grade 1−3,83/93 = 89.3% CRS grade 4−5, 10/93 = 10.7%.	NA	Overall CRS, 93/133 = 70% AKI with CRS, 3/93 = 3%	NA
Maude *et al.*S8	2018	11 yr (3−23 yr)	B- ALL	75	Needing RRT	AKI needing RRT, 7/75 = 9%	NA	CRS grade 1 and 2, 23/58 = 40% CRS grade 3, 16/58 = 28% CRS grade 4, 19/58 = 32%	NA	Overall CRS, 58/75 = 77% AKI with CRS needing RRT, 7/58 = 12%	Overall 12- mo mortality, 19/75 = 25%
Rives *et al.*S9	2018	Two groups: 1) <18 yr) (median = 10 yr, range 3−17 yr) 2) 18−25 yr (median 20 yr, range 18−25 y)	B- ALL	<18 yr, n = 84 18−25yr, n = 20	Needing RRT Needing RRT	NA NA	NA NA	CRS > grade 3, = 44% CRS >/ grade 3, 45%	NA	Overall CRS, 66/84 = 79% AKI/RRT with CRS, 7/66 = 11% Overall CRS, 18/20 = 90% AKI/RRT with CRS, 4/18 = 22%	Overall mortality >30 days, 21/84 = 25% Overall mortality, >30 days 8/20 = 40%
Hartsell *et al.*S10	2019	<25 yr (no range mentioned)	ALL DLDCL AML	40	Not defined	Overall AKI, 7/40 = 17.5%	NA	NA	NA	NA	Overall mortality in-hospital, =3/40 = 7.5%
Hartsell *et al.*S11	2019	>18 yr (no range mentioned)	B Cell Lymphoma, MM, Follicular lymphoma, ALL	735	Not defined	Overall AKI, 110/735 = 15%	NA	NA	NA	NA	Overall-in hospital mortality, 30/735 = 4%
TalleurS12	2019	<21 yr (no range mentioned)	Refractory ALL	5	Grade 3 AKI	Grade 3 AKI, 1/5 = 20%	NA	Grade 1 CRS, 1/1 = 100%. No mention of other grades	NA	Overall CRS, 1/5 = 20% CRS with AKI, 1/1 = 100%	NA
Myers *et al.*S13	2019	11 yr (range 1.4−29.1 yr)	Refractory B ALL	125	KDIGO	Overall AKI, = 26/125 (21.0%; 95% CI = 14.5−28.9), Severe AKI (KDIGO stage 2 and 3) 15 patients (12%; 95% CI = 7.3− 19.1), 3 patients (2.4%; 95% CI = 0.7−7.3) required RRT.	Patients with CRS had a 4.9 times greater risk of developing AKI (95% CI = 2.4− 9.9; *P* < 0.001) and a 10.3 times greater risk of developing severe AKI (95% CI = 3.1− 4.3; *P* < 0.001)	NA	NA	Overall CRS, 100/125 = 80%	NA
Metwally *et al.*S14	2019	Adults (age not defined)	DLBCL	N = 58	Rise in creatinine >0.3 above baseline	Overall AKI, 19/58 = 33%	NA	NA	NA	NA	AKI mortality at 6 months, 47% compared to 13% in non-AKI patients (*P* = 0.008)
Myers *et al.*S15	2019	12 yr (range 1.4− 29.1 yr)	ALL	Total N = 213, ICU admission, n = 49	Needing RRT	AKI needing RRT among ICU-admitted patients, 4/213 = 1.9%	NA	NA	NA	NA	Overall 30-day mortality, 2/213 = 1%
Harris *et al.*S16	2020	>18 yr	DLBCL	1570	Not defined	Overall AKI, 247/1570 = 15.7%	NA	NA	NA	NA	Overall mortality, 30/1570 = 1.5% (in-hospital mortality) No AKI mortality reported
Hiramatsu *et al.*S17	2020	Range 5−24 yr	B-ALL	6	Needing RRT	AKI needing RRT, 2/6 = 33%	NA	CRS grade 3, 2/5 = 40% CRS grade 4, 3/5 = 60%	NA	Overall CRS, 5/6 = 83% AKI with CRS, 2/5 = 40%	Overall 12-mo mortality, 2/6 = 33% No AKI mortality defined
Gutgarts *et al.*S18	2020	63 yr (range 19−86 yr)	DLBCL, NHL	46	KDIGO	Overall AKI, 14/46 = 30% (95% CI = 16.9%− 43.9%) Grade 1 AKI, 10/46 = 22% (95% CI = 9.7%− 33.8%) Grade 2, 2/46 = 4.5% Grade 3, 1/46 = 2.17% Grade 2/3 AKI, 8% (95% CI = 4%− 17%) incidence of grade 2−3 AKI No patients needed RRT	CRS, 11/14 = 79%	CRS grade 1 and 2, 31/37 = 84% CRS grade 3 and 4, 6/37 = 16%	Median GFR, 88 ml/min per 1.73 m^2^ (range, 36−160 ml/min per 1.73 m^2^)	Overall CRS, 37/46 = 80% (95% CI = 66% to 90.5%) CRS with AKI, 10/14 = 79%	30-day AKI mortality, 2/14 = 14%
Gupta *et al.*S19	2020	60 yr (mean age 60 ± 13 yr)	DLBCL	78	KDIGO	Overall AKI, 15/78 = 19% Stage 1, 7/78 = 9% Stage 2, 2/78 = 3% Stage 3, 6/78 = 6% AKI requiring RRT, 3/78 = 4%	Decreased kidney perfusion, 8 = 8/15 = 53% (CRS = 7/8) ATN, 6 = 6/15 = 40% (CRS = 6/6) Post- obstruction, 11/15 = 7%	CRS Grade 0, 12/66 = 18%. CRS grade 1, 28/66 = 42.4% CRS grade 2, 28/66 = 42.4% CRS grade 3, 8/66 = 12% CRS grade 4, 2/66 = 3%	109+/17 ml/min per 1.73 m^2^	Overall CRS, 66/78 = 85% CRS with AKI, 13/15 = 86%	Overall 6-mo mortality, 11/78 = 14% 6-mo AKI mortality, 5/15= 33%
Qu *et al.*S20	2020	48 yr (range 41−58 yr)	DLBCL	10 (C-CAR-T, n = 4) (R- CAR-T, n = 6)	Not defined	Overall AKI, 3/10 = 30 %	NA	CRS grade 3−5 (C-CAR-T), 4/10 = 40% CRS grade 1−2 (R-CAR-T), 6/10 = 60%	NA	Overall CRS, 10/10 = 100%	NA
ValadeS21	2020	56 yr (median (27−65 yr)	DLBCL ALL	41	AKI needing RRT	Overall AKI, 2/41 = 5%	NA	NA	NA	Overall CRS, 39/41 = 95%	NA
Lee *et al.*S22	2020	Mean age 60 yr (SD 18 yr)	DLBCL	37	AKI ≥1.5-fold rise in sCr from baseline	Overall AKI, 2/37 = 5% Stage 3 AKI, 2/37 = 5% AKI requiring RRT, 0/37 = 0%	NA	CRS grade 1, 15/20 = 75%. Grade 2, 4/20 = 20% Grade 3 and 4, 0 Grade 5, 1/20 = 5%	Baseline creatinine = 0.54 mg/dl	Overall CRS, 20/37 = 54%	Overall mortality, 5/37 = 14% AKI mortality,= 2/37 = 5%

AKI, acute kidney injury; ALL, acute lymphoblastic leukemia; AML, acute myeloid leukemia; B-NHL, B-cell non-Hodgkin’s lymphoma; CLL, chronic lymphoblastic leukemia; CRS, cytokine release syndrome; DLBCL, diffuse large B-cell lymphoma; GFR, glomerular filtration rate; ICU, intensive care unit; KDIGO, Kidney Disease: Improving Global Outcomes; NA, not available; NHL, non-Hodgkin's lymphoma; MM, multiple myeloma; RRT, renal replacement therapy; sCr, serum creatinine.

### Incidence of AKI Among Patients Treated With CAR-T Cell Therapies

**Figure 2:**
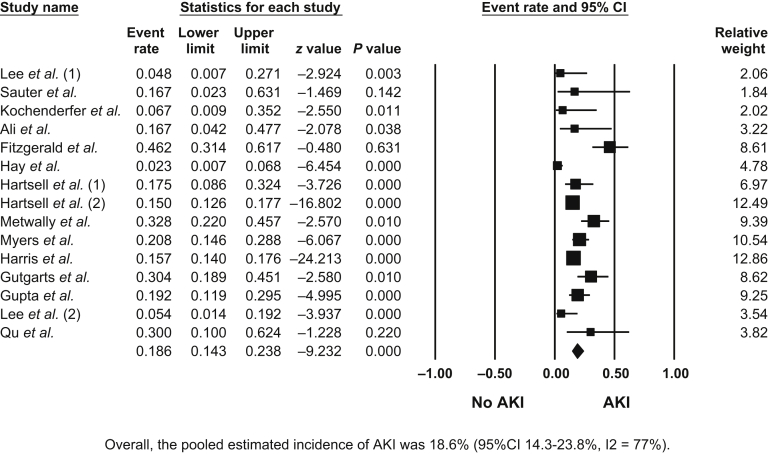
Incidence of acute kidney injury (AKI) among patients treated with chimeric antigen receptor T-cell (CAR-T) therapies. CI, confidence interval.

Overall, the pooled estimated incidence of AKI among patients treated with CAR-T therapies was 18.6% (95% CI = 14.3%−23.8%, I2 = 77%) (Figure 2), and the pooled estimated incidence of AKI-RRT was 4.4% (95% CI 2.1%−8.9%, I2 = 61%) (Supplementary Figure S1).

### Incidence of AKI Among Adults

Subgroup analysis among adults treated with CAR-T therapies resulted in the pooled estimated incidence of AKI at 17.0% (95% CI = 12.8%−22.2%, I2 = 73%) (Supplementary Figure S2), and the incidence of AKI-RRT was 2.9% (95% CI = 0.9%−9.4%, I2 = 41%) (Supplementary Figure S3). Upon analyzing the results using Kidney Disease: Improving Global Outcomes (KDIGO) criteria, we report that the pooled estimated AKI incidence among adults was 24.1% (95% CI = 14.9%−36.5%, I2 = 49%) (Supplementary Figure S4). The incidence of AKI among adults on CAR-T therapies was slightly higher by KDIGO criteria as compared to overall AKI incidence.

### Incidence of AKI Among Pediatric and Young Adult Patients

Among pediatric and young adult patients, the pooled estimated incidence of AKI was 22.5% (95% CI = 11.1%−40.1%, I2 = 79%) (Supplementary Figure S5) and AKI-RRT was 6.0% (95% CI = 2.2%−15.5%, I2 = 72%) (Supplementary Figure S6). We noticed higher rates of AKI and AKI-RRT among pediatric and young adults than reported among adults. In addition, higher rates of AKI were noted among pediatric and young adults even when analyzed using KDIGO criteria, with an estimated incidence of 31.7% (95% CI = 12.7%−59.6%, I2 = 89%) (Supplementary Figure S7).

### Incidence of CRS Among Patients With CAR-T Therapies

Among patients on CAR-T therapies, the estimated CRS incidence in all included studies was 75.4% (95% CI = 66.6%−82.4%, I2 = 71%) (Supplementary Figure S8). Upon analyzing the relationship between CRS severity and incidence of AKI-RRT, we found a strong correlation between them (slope = +0.0413, *P* = 0.01) (Supplementary Figure S9).

### Publication Bias

Funnel plots (Supplementary Figures S10−S13) and Egger regression asymmetry tests were performed to evaluate for publication bias. We found no significant publication bias in the meta-analysis evaluating AKI incidence (*P* ≥ 0.05 for all analysis).

## Discussion

In the current era, the utility of CAR-T therapies has extended widely among patients with various cancers. In our study, the pooled incidence of any AKI among all patients with CAR-T therapies was 19%, and the incidence of AKI-RRT was 4%. Upon analyzing studies that defined AKI by KDIGO criteria, the pooled incidence of AKI among adults was slightly higher at 24%. This observation could be secondary to using a definition with a higher sensitivity in identifying patients with AKI. Interestingly, most patients experienced only mild AKI, with a rise in serum creatinine ≥0.3 mg/dl to1.5 times baseline creatinine, and a smaller proportion of patients reached AKI stage 3 or AKI-RRT. Our findings are in concordance with the results of the largest published studies on AKI incidence among patients with CAR-T therapies.^4^^,^S14^,^ S18^,^ S19

We found that the pooled incidence of CRS among patients with CAR-T therapies was 75%, which is in agreement with major published studies.S1^,^S3^,^S6−S9^,^S13^,^S17−S20 Our analysis reports a strong correlation between AKI-RRT incidence and severe CRS. In addition, our study indicated a higher incidence of AKI and AKI-RRT in the pediatric population than in older adults (22.5% vs. 17% and 6% vs. 2.9%, respectively). The current observation could be secondary to increased CRS incidence among the pediatric population as reported by Lee *et al.* and Maulde *et al.*S3^,^S8 The underlying mechanisms for a higher incidence of CRS among the pediatric population are unclear. However, it is hypothesized to be related to a higher dose, excessive tumor burden, and association of ALL with higher blast count leading to increased CRS risk,^5^ and, finally, immaturity of the immune system among pediatric patients and young adults.

Our study has several strengths. It is the first systematic review looking at the pooled incidence of AKI among adults and pediatric subgroups receiving CAR-T therapies and reporting a correlation between incidence of AKI- RRT and severe CRS. However, our systematic review also has a few limitations. We included a few retrospective cohort studies with smaller sample sizes that reported associations rather than a causal relationship between AKI incidence and CAR-T cell therapies. There is modest heterogeneity among various studies in defining AKI. In addition, we do not have data on baseline kidney function except for a few studiesS18^,^S19 that included patients with median and mean baseline GFR >60 ml/min per m^2^ respectively.

In conclusion, we report that AKI incidence among patients with CAR-T therapies varies among pediatric and adult cohorts. We additionally report a strong correlation between the severity of CRS and AKI-RRT. As AKI-RRT is associated with higher mortality and morbidity, it would be helpful to undertake additional preventive strategies along with the addition of tocilizumab in patients at high risk for severe CRS to mitigate the risk of AKI. Future research on identifying models or biomarkers that could help to predict AKI among patients on CAR-T therapies and the impact of preventive measures on the incidence of AKI, AKI-RRT, and severe CRS is warranted.

## Disclosure

All the authors declared no competing interests.

## Supplementary Material

**Supplementary Methods**

**Figure S1.** Incidence of severe AKI or AKI-RRT among patients on CAR-T therapies.

**Figure S2.** Incidence of overall AKI among adults on CAR-T therapies.

**Figure S3.** Incidence of AKI- RRT among adults on CAR-T therapies.

**Figure S4.** Incidence of overall AKI (only KDIGO definition) among adults on CAR-T therapies.

**Figure S5.** Incidence of overall AKI among pediatric patients and young adults on CAR-T therapies.

**Figure S6.** Incidence of severe AKI- RRT among pediatric patients and young adults.

**Figure S7.** Incidence of overall AKI (only KDIGO definition) among pediatric patients and young adults.

**Figure S8.** Overall incidence of CRS among patients on CAR-T therapies.

**Figure S9.** Relation between CRS severity of incidence of AKI-RRT among patients on CAR-T therapies.

**Figure S10.** Funnel plot of adults with AKI on CAR-T therapy.

**Figure S11.** Funnel plot of adults with severe AKI-RRT therapy.

**Figure S12.** Funnel plot of pediatric patients and young adults with AKI on CAR-T therapy.

**Figure S13.** Funnel plot of pediatric and young adult patients with severe AKI-RRT on CAR-T therapy.

**Supplementary References**
