# Supplementary material for: Systematic Review of Risk factors and Incidence of Acute Kidney Injury Among Patients Treated with CAR-T Cell Therapies
Source: Kidney Int Rep. 2021 Feb 17;6(5):1416–22. doi: 10.1016/j.ekir.2021.02.013 (PMC8116758; doi:10.1016/j.ekir.2021.02.013)
Supplement: Supplementary File (PDF) [file mmc1.pdf]

Supplementary Data

Methods

Search Strategy

We followed the PRISMA [S23] (Preferred Reporting Items for Systematic Reviews and Meta-Analysis) statement in conducting the current systematic review. EMBASE, Cochrane, and Ovid MEDLINE Databases were systematically searched from database inception through May 2020. We additionally performed an updated literature search in September 2020 to include all relevant studies. The protocol for this meta-analysis is registered with PROSPERO; no. CRD42020184601. Three investigators (S.K., T.P., and S.M) performed an independent literature search. Kappa coefficient of agreement for the investigators was 0.86. Information on the detailed search strategy is provided below. Language restriction was not applied. Potentially related studies were manually reviewed using the references.

Study Selection

Observational studies and clinical trials providing 95% confidence intervals (CI) data on the incidence of AKI, CRS and severe CRS (CRS grade ≥ 3) among adults and pediatric patients receiving CAR-T cells were included in the meta-analysis. Follow up period among these studies ranged from 30 days to one year. We also included cohort studies with smaller sample size to yield more comprehensive outcomes. We further excluded case reports, animal studies, review articles and correspondence. Three investigators (S.K., T.P., and S.M) independently reviewed the titles and abstracts of the studies identified in the search based on inclusion and exclusion criteria. A third reviewer (S.H.) solved inconsistencies that occurred among two investigators by collective agreement. Among included studies, there are modest heterogenicities in AKI definition ranging from criteria of doubling of serum creatinine to 25% increase in serum creatinine from baseline. Additionally, KDIGO definitions [S24] of AKI were used for subgroup analysis. Severe AKI defines as AKI stage 3 or AKI requiring RRT.

Data Collection

The collected data from individual studies included the title, name of authors, publication year, age group/median age, type of cancer, AKI definition, the incidence of AKI and severe AKI requiring RRT, CRS incidence, grades of CRS.

Statistical analysis

Adjusted point estimates of included studies were incorporated by the generic inverse variance method of DerSimonian-Laird, which assigned individual study weight based on its variance [S25]. Due to the probability of between-study variance, we applied a random-effects model to pool outcomes of interest, including AKI incidence. We used Cochran's Q test ($p<0.05$ for a statistical significance) and I^2 statistic (≤25% represents insignificant heterogeneity, 26% to 50% represents low heterogeneity, 51% to 75% represents moderate heterogeneity, and ≥75% represents high heterogeneity) to assess statistical heterogeneity [S26]. Publication bias was analyzed by Funnel plots and Egger test [S27]. Meta-analysis was performed using Comprehensive Meta-Analysis software version 3.3.070 (Biostat Inc, New Jersey, USA). The data for this meta-analysis are publicly available through the Open Science Framework (URL: https://osf.io/872am/).

Search strategies.

Database: Ovid MEDLINE(R) and Epub Ahead of Print, In-Process & Other Non-Indexed Citations, Daily and Versions(R) (312 articles)

Search Strategy:

----

1 car t-cell therapy.mp.

2 exp car t-cell therapy/

3 chimeric antigen receptor immunotherapy.mp.

4 car-t.mp.

5 car t.mp.

6 chimeric antigen receptor t-cell.mp.

7 car t-cell.mp.

8 1 or 2 or 3 or 4 or 5 or 6 or 7

9 kidney disease.mp.

10 exp kidney disease/

11 exp acute kidney failure/

12 acute kidney failure.mp.
13 exp acute renal failure/
14 acute renal failure.mp.
15 acute tubular necrosis.mp.
16 acute kidney injury.mp.
17 exp acute kidney injury/
18 AKI.mp.
19 acute kidney disease.mp.
20 dialysis.mp.
21 hemodialysis.mp.
22 peritoneal dialysis.mp.
23 renal replacement therapy.mp
24 ICU.mp.
25 intensive care unit.mp.
26 9 or 10 or 11 or 12 or 13 or 14 or 15 or 16 or 17 or 18 or 19 or 20 or 21 or 22 or 23 or 24 or 25
27 8 and 26

****

EMBASE and Cochrane Databases

SEARCH QUERY **(169 articles)**

('chimeric antigen receptor immunotherapy' OR 'car-t' OR 'chimeric antigen receptor t-cell' OR 'car t-cell' OR 'car t-cell therapy') AND ('kidney disease' OR 'acute kidney failure' OR 'acute renal failure' OR 'acute kidney tubule necrosis' OR 'acute tubular necrosis' OR 'acute kidney injury' OR 'aki' OR 'acute kidney injury network criteria' OR 'acute kidney injury network' OR 'acute kidney disease' OR 'acute kidney injury network classification' OR 'renal replacement therapy' OR dialysis OR hemodialysis OR 'peritoneal dialysis' OR 'icu' OR 'intensive care unit').

Supplemental figures:

Figure S1. Incidence of severe AKI or AKI – RRT among patients on CAR-T therapies.

Study name	Study Year	Event rate	Lower limit	Upper limit	Z-Value	p-Value	Relative weight
Locke et al	2017	0.143	0.020	0.581	-1.659	0.097	7.36
Hay et al	2017	0.008	0.001	0.051	-4.864	0.000	7.98
Maude et al	2018	0.093	0.045	0.183	-5.728	0.000	14.66
Talleur et al	2019	0.200	0.027	0.691	-1.240	0.215	7.07
Myers et al (1)	2019	0.024	0.008	0.072	-6.340	0.000	12.42
Myers et al (2)	2019	0.019	0.007	0.049	-7.837	0.000	13.38
Hiramatsu et al	2020	0.333	0.084	0.732	-0.800	0.423	9.26
Gupta et al	2020	0.038	0.012	0.113	-5.467	0.000	12.36
Gutgarts et al	2020	0.011	0.001	0.149	-3.188	0.001	5.17
Fitzgerald et al	2017	0.013	0.001	0.171	-3.070	0.002	5.17
Lee et al (2)	2020	0.013	0.001	0.178	-3.033	0.002	5.16
		0.044	0.021	0.089	-7.999	0.000	

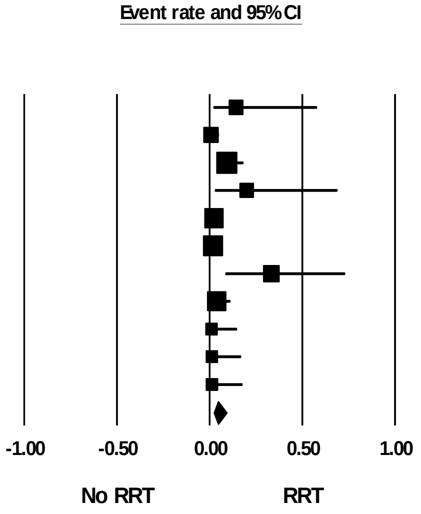

The pooled estimated incidence of severe AKI or AKI requiring RRT was 4.4% (95%CI 2.1-8.9%, I2 = 61%).

Figure S2. Incidence of overall AKI among adults on CAR-T therapies

Study name	Event rate	Lower limit	Upper limit	Z-Value	p-Value	Relative weight
Sauter et al	0.167	0.023	0.631	-1.469	0.142	2.14
Kochenderfer et al	0.067	0.009	0.352	-2.550	0.011	2.37
Ali et al	0.167	0.042	0.477	-2.078	0.038	3.88
Hay et al	0.023	0.007	0.068	-6.454	0.000	5.96
Hartsell et al (2)	0.150	0.126	0.177	-16.802	0.000	19.04
Metwally et al	0.328	0.220	0.457	-2.570	0.010	13.14
Gutgarts et al	0.304	0.189	0.451	-2.580	0.010	11.82
Gupta et al	0.192	0.119	0.295	-4.995	0.000	12.89
Harris et al	0.157	0.140	0.176	-24.213	0.000	19.81
Lee (2)	0.054	0.014	0.192	-3.937	0.000	4.29
Qu et al	0.300	0.100	0.624	-1.228	0.220	4.66
	0.170	0.128	0.222	-9.370	0.000	

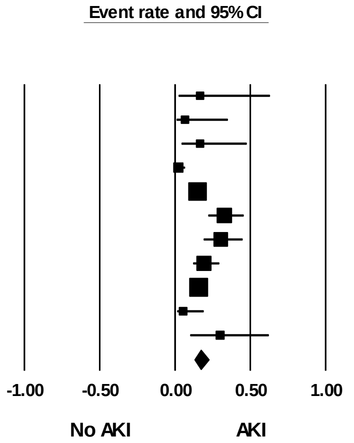

Overall, the pooled estimated incidence of AKI among adults was 17.0% (95%CI 12.8-22.2%, I2 = 73%).

Figure S3. Incidence of AKI- RRT among adults on CAR-T therapies

Study name	Study Year	Event rate	Lower limit	Upper limit	Z-Value	p-Value	Relative weight
Locke et al	2017	0.143	0.020	0.581	-1.659	0.097	18.09
Hay et al	2017	0.008	0.001	0.051	-4.864	0.000	20.16
Gupta et al	2020	0.038	0.012	0.113	-5.467	0.000	38.45
Gutgarts et al	2020	0.011	0.001	0.149	-3.188	0.001	11.66
Lee et al (2)	2020	0.013	0.001	0.178	-3.033	0.002	11.63
		0.027	0.010	0.073	-6.757	0.000	

Event rate and 95%CI

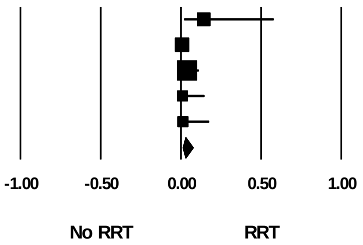

Estimated incidence of AKI- RRT was 2.9% (95%CI 0.9-9.4%, I2 = 41%).

Figure S4. Incidence overall AKI (only KDIGO definition) among adults on CAR-T therapies

Study name	Event rate	Lower limit	Upper limit	Z-Value	p-Value	Relative weight
Gutgarts et al	0.304	0.189	0.451	-2.580	0.010	47.28
Gupta et al	0.192	0.119	0.295	-4.995	0.000	52.72
	0.241	0.149	0.365	-3.778	0.000	

Event rate and 95%CI

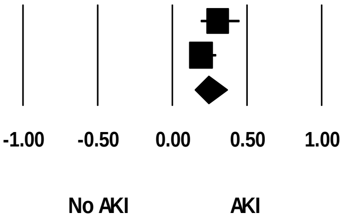

Pooled estimated incidence of AKI was 24.1% (95%CI 14.9-36.5%, I2 = 49%).

Figure S5. Incidence overall AKI among pediatrics and young adults on CAR-T therapies

Study name	Event rate	Lower limit	Upper limit	Z-Value	p-Value	Relative weight
Lee et al (1)	0.048	0.007	0.271	-2.924	0.003	11.67
Fitzgerald et al	0.462	0.314	0.617	-0.480	0.631	29.49
Hartsell et al (1)	0.175	0.086	0.324	-3.726	0.000	26.50
Myers et al	0.208	0.146	0.288	-6.067	0.000	32.34
	0.225	0.111	0.401	-2.896	0.004	

Event rate and 95% CI

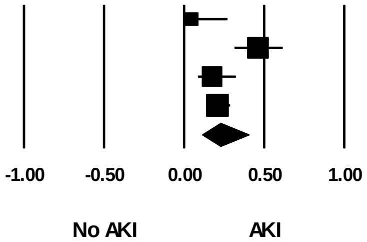

Overall, the pooled estimated incidence of AKI was 22.5% (95%CI 11.1-40.1%, I2 = 79%).

Figure S6. Incidence severe AKI- RRT among pediatric and young adults

Study name	Study Year	Event rate	Lower limit	Upper limit	Z-Value	p-Value	Relative weight
Fitzgerald et al	2017	0.013	0.001	0.171	-3.070	0.002	9.19
Maude et al	2018	0.093	0.045	0.183	-5.728	0.000	22.53
Talleur et al	2019	0.200	0.027	0.691	-1.240	0.215	12.19
Myers et al (1)	2019	0.024	0.008	0.072	-6.340	0.000	19.71
Myers et al (2)	2019	0.019	0.007	0.049	-7.837	0.000	20.95
Hiramatsu et al	2020	0.333	0.084	0.732	-0.800	0.423	15.43
		0.060	0.022	0.155	-5.104	0.000	

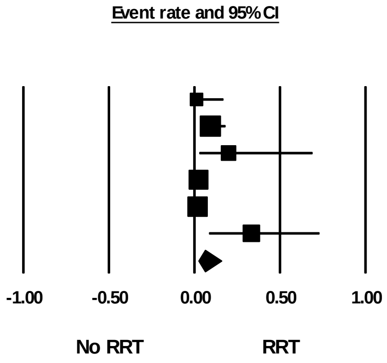

Estimated incidence of severe AKI -RRT was 6.0% (95%CI 2.2-15.5%, I2 = 72%).

Figure S7. Incidence overall AKI (only KDIGO definition) among pediatric and young adults

Study name	Event rate	Lower limit	Upper limit	Z-Value	p-Value	Relative weight
Fitzgerald et al	0.462	0.314	0.617	-0.480	0.631	48.05
Myers et al	0.208	0.146	0.288	-6.067	0.000	51.95
	0.317	0.127	0.596	-1.301	0.193	

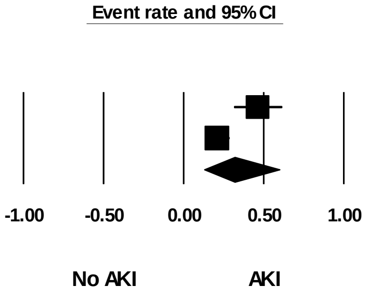

The estimated incidence of AKI was 31.7% (95%CI 12.7-59.6%, I2 = 89%).

Figure S8. Overall Incidence of CRS among patients on CAR-T therapies

Study name	Event rate	Lower limit	Upper limit	Z-Value	p-Value	Relative weight
Sauter et al	0.333	0.084	0.732	-0.800	0.423	4.16
Lee et al (1)	0.762	0.540	0.897	2.270	0.023	7.26
Ali et al	0.333	0.131	0.624	-1.132	0.258	6.19
Locke et al	0.857	0.419	0.980	1.659	0.097	3.05
Hay et al	0.699	0.616	0.771	4.462	0.000	11.08
Maude et al	0.773	0.665	0.854	4.450	0.000	10.13
Talleur et al	0.200	0.027	0.691	-1.240	0.215	2.90
Myers et al	0.800	0.721	0.861	6.200	0.000	10.73
Hiramatsu et al	0.833	0.369	0.977	1.469	0.142	2.99
Gutgarts et al	0.804	0.665	0.895	3.804	0.000	8.95
Gupta et al	0.846	0.748	0.911	5.432	0.000	9.67
Qu et al	0.955	0.552	0.997	2.103	0.035	1.91
Valade et al	0.951	0.825	0.988	4.097	0.000	5.18
Fitzgerald et al	0.923	0.787	0.975	4.135	0.000	6.31
Lee (2)	0.541	0.381	0.692	0.493	0.622	9.47
	0.754	0.666	0.824	5.120	0.000	

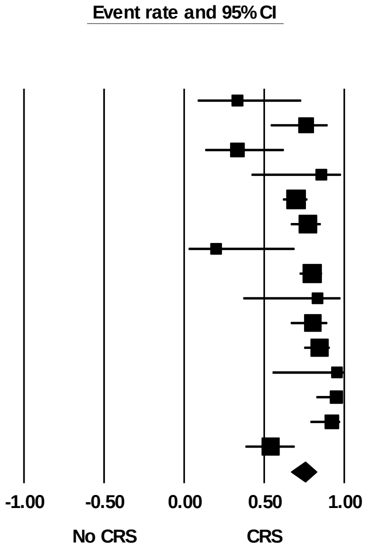

Estimated incidence of CRS in all included studies was 75.4% (95%CI 66.6-82.4%, I2 = 71%).

Figure S9. Relation between CRS severity and Incidence of AKI-RRT among patients on CAR-T therapies.

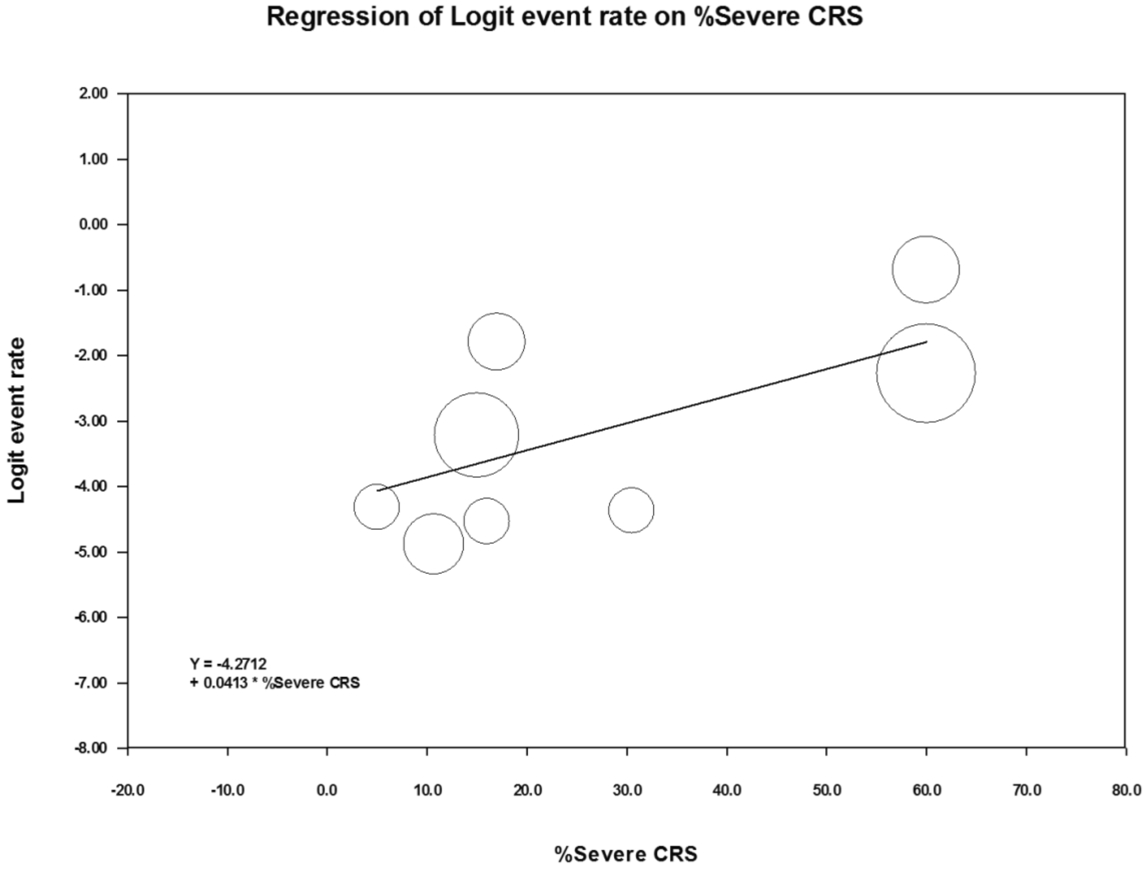

Severity of CRS was significantly correlated with the incidence of severe AKI requiring RRT (slope = +0.0413, p = 0.01).

Figure S10. Funnel plot adults with AKI- CAR-T therapy

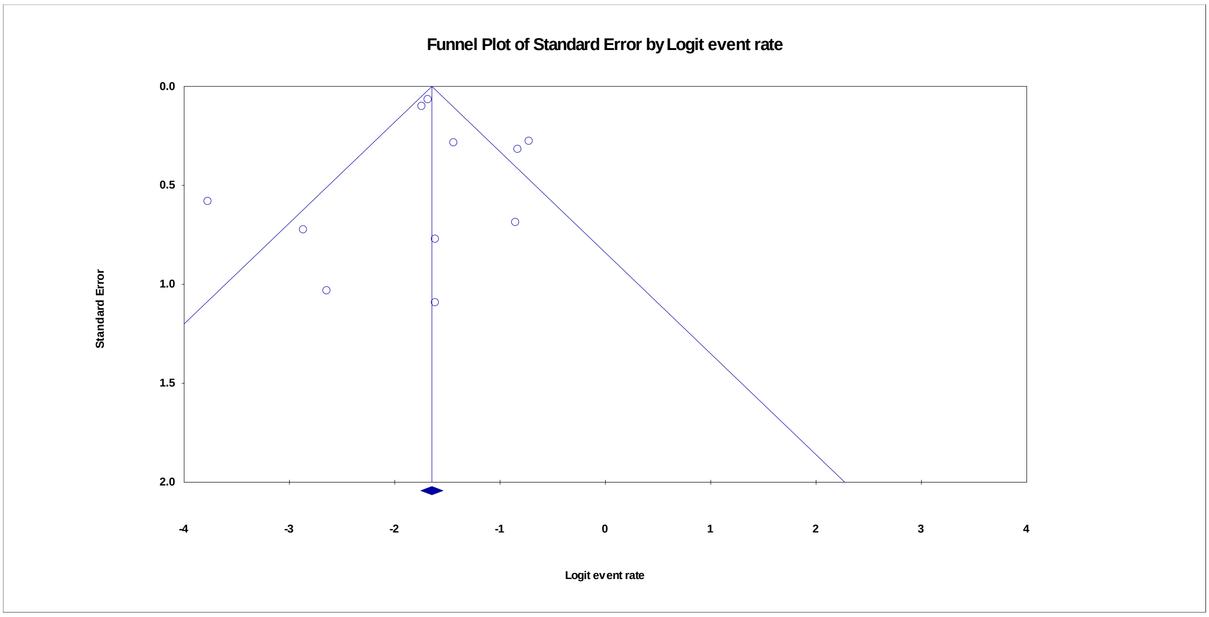

Figure S11. Funnel plot adults with severe AKI- RRT on CAR-T therapy

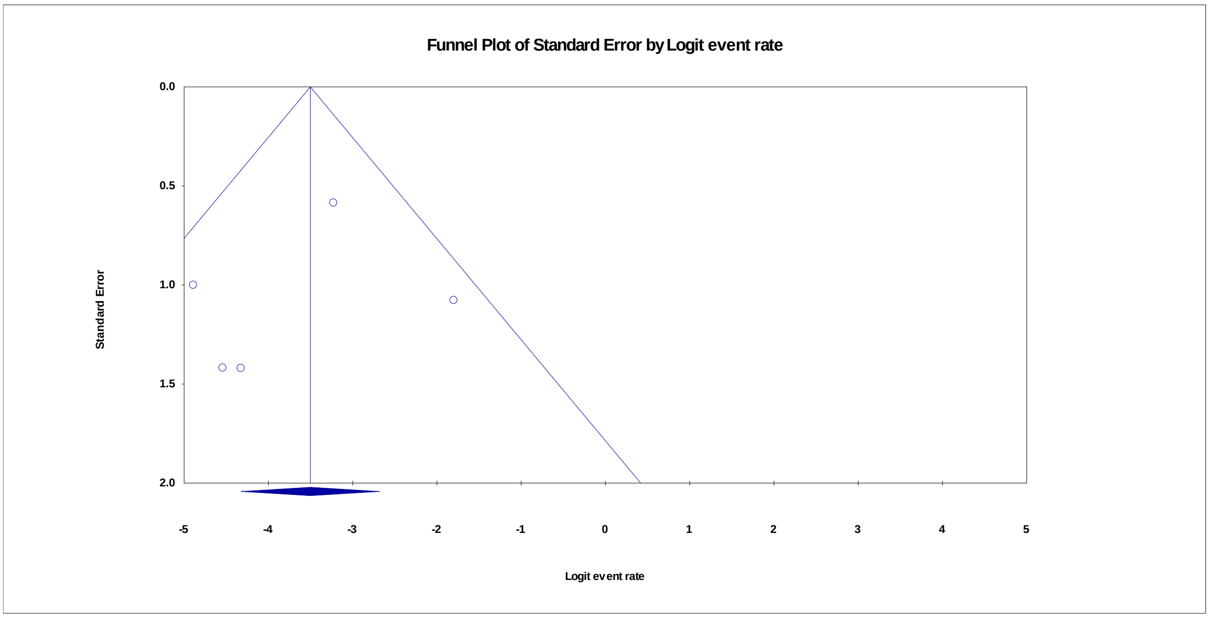

Figure S12. Funnel plot pediatric and young adults with AKI on CAR-T therapy

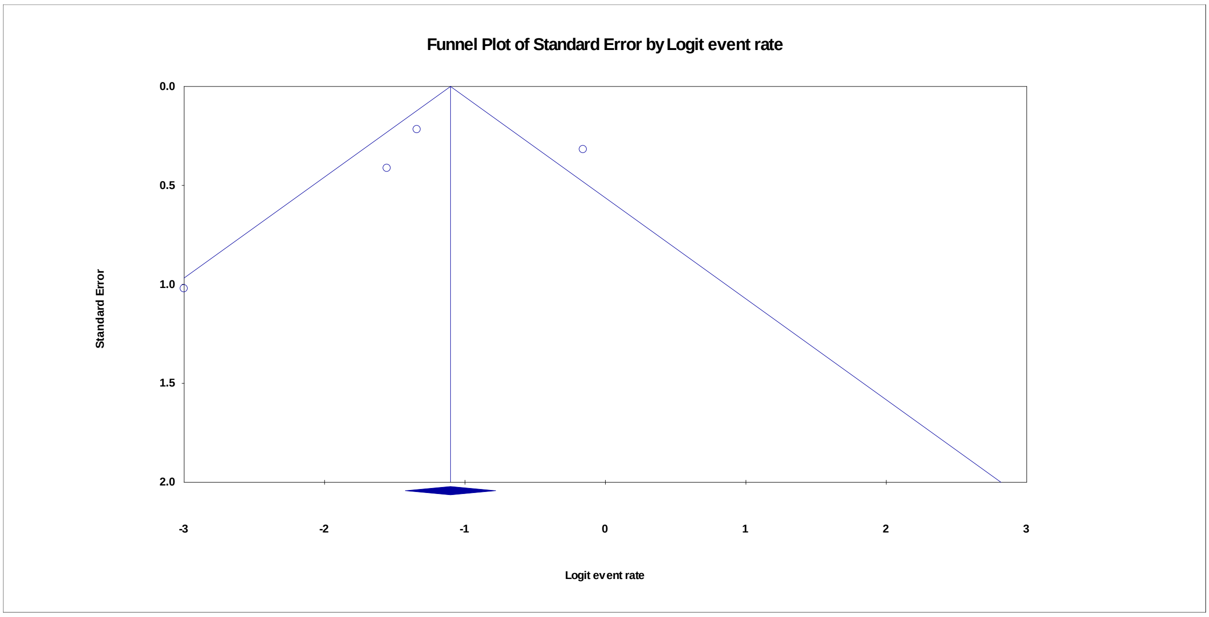

Figure S13. Funnel plot pediatric and young adults with severe AKI – RRT on CAR-T therapy.

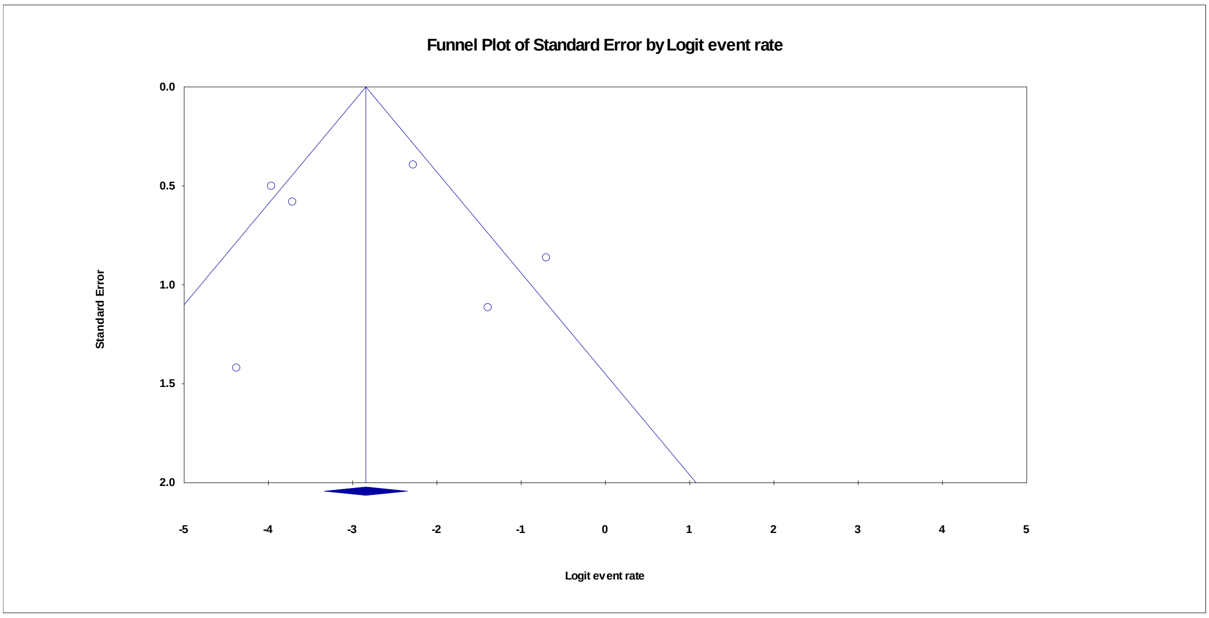

Supplementary References

S1. Fitzgerald JC, Weiss SL, Maude SL*, et al.* Cytokine Release Syndrome After Chimeric Antigen Receptor T Cell Therapy for Acute Lymphoblastic Leukemia. *Crit Care Med* 2017; **45:** e124-e131.

S2. Sauter CS, Riviere I, Bernal YJ*, et al.* Interim Safety Analysis: a Phase I Trial of High Dose Therapy and Autologous Stem Cell Transplantation Followed By Infusion of Chimeric Antigen Receptor Modified T-Cells (19-28z CAR-T) Directed Against CD19+ B-Cells for Relapsed and Refractory Aggressive B Cell Non-Hodgkin Lymphoma (B-NHL). *Blood* 2014; **124:** 677-677.

S3. Lee DW, Kochenderfer JN, Stetler-Stevenson M*, et al.* T cells expressing CD19 chimeric antigen receptors for acute lymphoblastic leukaemia in children and young adults: a phase 1 dose-escalation trial. *Lancet* 2015; **385:** 517-528.

S4. Kochenderfer JN, Dudley ME, Kassim SH*, et al.* Chemotherapy-refractory diffuse large B-cell lymphoma and indolent B-cell malignancies can be effectively treated with autologous T cells expressing an anti-CD19 chimeric antigen receptor. *J Clin Oncol* 2015; **33:** 540-549.

S5. Ali SA, Shi V, Maric I*, et al.* T cells expressing an anti-B-cell maturation antigen chimeric antigen receptor cause remissions of multiple myeloma. *Blood* 2016; **128:** 1688-1700.

S6. Locke FL, Neelapu SS, Bartlett NL*, et al.* Phase 1 Results of ZUMA-1: A Multicenter Study of KTE-C19 Anti-CD19 CAR T Cell Therapy in Refractory Aggressive Lymphoma. *Mol Ther* 2017; **25:** 285-295.

S7. Hay KA, Hanafi LA, Li D*, et al.* Kinetics and biomarkers of severe cytokine release syndrome after CD19 chimeric antigen receptor-modified T-cell therapy. *Blood* 2017; **130:** 2295-2306.

S8. Maude SL, Laetsch TW, Buechner J*, et al.* Tisagenlecleucel in Children and Young Adults with B-Cell Lymphoblastic Leukemia. *New England Journal of Medicine* 2018; **378:** 439-448.

S9. Susana Rives MWB, André Baruchel, Jochen Buechner, Hidefumi Hiramatsu, Paul L. Martin, Francoise Mechinaud, Gary Douglas Myers, Eneida Nemecek, Michael A. Pulsipher, Michael R. Verneris, Rajen Mody, Lamis K. Elderjou, Yelena Lalazar, Lan Yi, Stephan A. Grupp. OUTCOMES OF YOUNG ADULT (≥ 18-25 YEARS) AND PEDIATRIC (<18 YEARS) PATIENTS WITH RELAPSED/REFRACTORY ACUTE LYMPHOBLASTIC LEUKEMIA FOLLOWING TREATMENT WITH CHIMERIC ANTIGEN RECEPTOR (CAR) T-CELL THERAPY. *EHA Library* 2018.

S10. Hartsell A. Emerging Trends in Chimeric Antigen Receptor T-Cell Immunotherapy in Young Adults and Pediatric Patients from the Vizient Clinical Database. *Biology of Blood and Marrow Transplantation* 2019; **25:** S337.

S11. Hartsell A. Emerging Trends in Chimeric Antigen Receptor T-Cell Immunotherapy in Adults from the Vizient Clinical Database. *Biology of Blood and Marrow Transplantation* 2019; **25:** S336-S337.

S12. Talleur A, Qudeimat A, Lockey T*, et al.* Autologous CD19-CAR T-Cells for the Treatment of Acute Lymphoblastic Leukemia in Pediatric and Young Adult Patients: An initial Report from an Institutional Phase I/II Study. *Clinical Lymphoma Myeloma and Leukemia* 2019; **19:** S265.

S13. Myers RM, Fitzgerald J, Elgarten CW*, et al.* Acute Kidney Injury after Chimeric Antigen Receptor T-Cell Therapy for Pediatric Acute Lymphoblastic Leukemia. *Biology of Blood and Marrow Transplantation* 2019; **25:** S168-S169.

S14. Metwally Sea. Predictors of AKI in patients undergoing CART- Cell Therapy. *JASN abstract supplement 2019* 2019.

S15. Myers R, Fitzgerald J, DiNofia A*, et al.* Inpatient and Intensive Care Unit Resource Utilization after CD19-Targeted Chimeric Antigen Receptor T-Cell Therapy (CART19) for Pediatric Acute Lymphoblastic Leukemia (ALL). *Biology of Blood and Marrow Transplantation* 2020; **26:** S202-S203.

S16. Harris AH, Hohmann S, Shippey E*, et al.* Quality and Cost Outcomes in Chimeric Antigen Receptor T-Cell Immunotherapy in Adult Large B-Cell Cancer Patients from the Vizient Clinical Database. *Biology of Blood and Marrow Transplantation* 2020; **26:** S316-S317.

S17. Hiramatsu H, Adachi S, Umeda K*, et al.* Efficacy and safety of tisagenlecleucel in Japanese pediatric and young adult patients with relapsed/refractory B cell acute lymphoblastic leukemia. *Int J Hematol* 2020; **111:** 303-310.

S18. Gutgarts V, Jain T, Zheng J*, et al.* Acute Kidney Injury after CAR-T Cell Therapy: Low Incidence and Rapid Recovery. *Biol Blood Marrow Transplant* 2020; **26:** 1071-1076.

S19. Gupta S, Seethapathy H, Strohbehn IA*, et al.* Acute Kidney Injury and Electrolyte Abnormalities After Chimeric Antigen Receptor T-Cell (CAR-T) Therapy for Diffuse Large B-Cell Lymphoma. *Am J Kidney Dis* 2020; **76:** 63-71.

S20. Qu C, Ping N, Kang L*, et al.* Radiation Priming Chimeric Antigen Receptor T-Cell Therapy in Relapsed/Refractory Diffuse Large B-Cell Lymphoma With High Tumor Burden. *J Immunother* 2020; **43:** 32-37.

S21. S V. CAR-T cell therapy in ICU patients: A single-center experience. *Annals of Intensive Care (2020) 10 Supplement 1 Date of Publication: 2020* 2020.

S22. Lee M. Acute Kidney Injury After the CAR-T cell Therapy Tisagenlecleucel. *AJKD* 2020.

S23. Moher D, Shamseer L, Clarke M*, et al.* Preferred reporting items for systematic review and meta-analysis protocols (PRISMA-P) 2015 statement. *Systematic Reviews* 2015; **4:** 1.

S24. Khwaja A. KDIGO Clinical Practice Guidelines for Acute Kidney Injury. *Nephron Clinical Practice* 2012; **120:** c179-c184.

S25. DerSimonian R, Laird N. Meta-analysis in clinical trials. *Control Clin Trials* 1986; **7:** 177-188.

S26. Higgins JP, Thompson SG, Deeks JJ*, et al.* Measuring inconsistency in meta-analyses. *BMJ* 2003; **327:** 557-560.

S27. Easterbrook PJ, Berlin JA, Gopalan R*, et al.* Publication bias in clinical research. *Lancet* 1991; **337:** 867-872.
